# 

**Published:** 1904-01

**Authors:** 


THE AMERICAN

### Journal of Dental Science.

THIRD SERIES. D-5559

THE OLDEST DENTAL MAGAZINE IN THE WORLD.

VOL. XXXV. JANUARY, 1904. NO. 1.

EDITOR:
WM. GIRD BEECROFT, D. D. S., Madison, Wis.
ASSOCIATE EDITORS:
JOHN P. BUCKLEY, Ph. G., D. D. S., Chicago.
ALBERT H. KETCHAM, D. D. S., Denver, Colo.
EMORY A. BRYANT, D. D. S., Washington, D. C.
Published on the 10th day of each month.
Subscription Price, $1.00 per year.
Foreign Countries, $1.50 per year.
Address all communications, both business and editorial to the editor.
**Entered as Second Class Matter, Madison, Wis.**

